# Women’s views and experiences of augmentation of labour with synthetic oxytocin infusion. A protocol for a qualitative evidence synthesis.

**DOI:** 10.12688/hrbopenres.13467.2

**Published:** 2022-04-20

**Authors:** Silvia Alòs-Pereñíguez, Deirdre O'Malley, Deirdre Daly

**Affiliations:** 1 School of Nursing & Midwifery, Faculty of Health Sciences, Trinity College Dublin, Dublin, D02 T283, Ireland; 2 Nursing, Midwifery & Health Studies, Dundalk Institute of Technology, Dundalk, A91 K584, Ireland

**Keywords:** Birth experience, Oxytocin, Pregnancy, Labor, Obstetric, Qualitative systematic review, acceleration of labour

## Abstract

**Background:** Augmentation of labour (AOL) is the most common intervention to treat labour dystocia. Previous research reported extensive disparities in AOL rates across countries and institutions.  Despite its widespread use, women’s views on and experiences of intrapartum augmentation with infused synthetic oxytocin are limited.

**Methods:** A qualitative evidence synthesis on women’s views and experiences of AOL with synthetic oxytocin after spontaneous onset of labour will be conducted. Qualitative studies and studies employing a mixed methods design, where qualitative data can be extracted separately, will be included, as will surveys with open-ended questions that provide qualitative data. A systematic search will be performed of the databases: MEDLINE, CINAHL, EMBASE, PsycINFO, Maternity and Infant Care and Web of Science Core Collection from the date of inception. The methodological quality of included studies will be assessed using the Evidence for Policy and Practice Information and Co-ordinating Centre’s appraisal tool. A three-stage approach, coding of data from primary studies, development of descriptive themes and generation of analytical themes, will be used to synthesise findings. Confidence in findings will be established by the Grading of Recommendations Assessment, Development and Evaluation-Confidence in the Evidence from Reviews of Qualitative research.

**Discussion:** This qualitative evidence synthesis may provide valuable information on women’s experiences of AOL and contribute to a review of clinical practice guidelines for maternity care providers.

**PROSPERO registration:** CRD42021285252 (14/11/2021)

## Introduction

Augmentation of labour (AOL), the process of stimulating the uterus to increase the frequency, duration, and intensity of contractions after spontaneous onset of labour ( World Health Organization (WHO), 2014), has been identified as one of the most common obstetric interventions ( Miller *et al.*, 2016; Seijmonsbergen-Schermers *et al.*, 2020). Typically performed using a synthetic oxytocin infusion following artificial rupture of membranes, it is indicated in the management of labour dystocia, one of the main underlying reasons for performing caesarean sections ( Boyle *et al.*, 2013; Riddell *et al.*, 2017). The proportion of women who have their labour augmented ranges from 22% to 71% in nulliparous women, and from 7% to 38% in multiparous women in high income countries, even after adjusting by population characteristics ( Seijmonsbergen-Schermers *et al.*, 2020). Wide variation is also seen across maternity services within the same country, suggesting a potential misuse in clinical practice ( Helbig *et al.*, 2019; Seijmonsbergen-Schermers *et al.*, 2018).

While the main indication for AOL is for the management of labour dystocia ( Bugg *et al.*, 2013), the lack of consensus on the diagnostic criteria for labour dystocia has brought into question the robustness of its diagnosis ( Karaçam *et al.*, 2014; Neal *et al.*, 2015a; Neal *et al.*, 2015b; Oladapo *et al.*, 2018). In addition, there does not appear to be agreement on the regimens and doses of oxytocin that should be used ( Daly *et al.*, 2020). Two systematic reviews on AOL have demonstrated its effectiveness in accelerating labour progression, with a reduction of two hours in mean labour duration ( Bugg *et al.*, 2013; Kenyon *et al.*, 2013). However, data regarding women’s experiences of AOL in both reviews was limited and recent studies have shown that AOL has been associated with negative childbirth experiences ( Johansson & Finnbogadóttir, 2019; Nahaee *et al.*, 2020; Nystedt & Hildingsson, 2018).

Childbirth is a transcendental life experience that can lead to either positive feelings of empowerment and fulfilment or negative feelings of disappointment and fear, and can influence women’s decision towards having another baby or a desire for a caesarean section in future pregnancies ( Fuglenes *et al.*, 2011; Khajehei & Doherty, 2018; Larkin *et al.*, 2012; Nystedt & Hildingsson, 2014; Suwanrath *et al.*, 2021). Moreover, women with negative childbirth experiences are more vulnerable for developing serious mental health issues, including posttraumatic stress disorder ( Ertan *et al.,* 2021). The WHO recommendations on intrapartum care for a positive childbirth experience state that most women pursue a physiological birth and want to be involved in decision-making when medical interventions are needed ( WHO, 2018).

Women’s experiences and perspectives of AOL are not well understood. While there is a plethora of quantitative research on AOL, there are very few qualitative studies about women's experiences. This qualitative evidence synthesis (QES) aims to integrate the findings from studies reporting on women’s views and experiences of AOL with synthetic oxytocin after spontaneous onset of labour in order to deepen understanding and contribute to future reviews of clinical practice guidelines for maternity care providers.

## Protocol

### Inclusion criteria

We use the SPIDER (sample, phenomenon of interest, design, evaluation, and research type) tool ( Cooke *et al.*, 2012) to identify the key concepts for inclusion criteria in the QES.

- **S**ample: women of any age, parity, and cultural background who, after spontaneous onset of labour, underwent AOL with synthetic oxytocin.

- **P**henomenon of Interest: women’s views and experiences of AOL with synthetic oxytocin. Alternative terminology referring to AOL such as augmentation, acceleration or stimulation of labour has been considered to ensure a wide retrieval of data ( Table 1).

**Table 1.** Search terms.

**S**ample	women* OR woman* OR mother* OR parturit* OR matern* OR pregnan* OR nullipar* OR multipar* OR postnatal* OR perinatal* OR post-natal* OR peri-natal* OR childbirth* OR birth* OR postpartum*
**P**henomenon of **I**nterest	(augment* OR accelerat* OR stimulat* OR speed*) N4 (labor* OR labour* OR birth* OR parturit* OR childbirth* OR “child birth*”) OR oxitoc* OR oxytoc* OR ocytoc* OR ocitoc* OR pitocin OR syntocinon OR “oxytocin-induce*” OR “oxytocin prepar*”
**D**esign or **E**valuation	(“qualitativ*” OR “qualitative stud*” OR “qualitative analysis” OR “qualitative method*” OR “focus group*” OR interview* OR “triangulat*” OR “narrative*” OR “naturalistic inqur*” OR “feminist research*” OR “grounded theor*” OR “hermeneutic*” OR “phenomenol*” OR “ethnograph*” OR “ethnonurs*” OR “ethnological research” OR “ethnomethodolog*” OR “purposive sampl*” OR “theoretical sampl*” OR “thematic analysis” OR “content analysis” OR “discourse analysis” OR “action research” OR “participatory research” OR “constant comparative method” OR “mixed model” OR “mixed method*” OR “mixed design*” OR “multiple method*” OR multimethod* OR “open-ended question*” OR “open-ended survey*” OR “open-ended interview*”) OR (experien* OR view* OR percept* OR perceiv* OR attitud* OR believ* OR belief* OR perspective* OR opinion* OR express* OR thought* OR think* OR feel* OR reaction* OR emotion* OR comprehen* OR understand* OR stance* OR “personal valu*” OR awar* OR approach* OR “self report*” OR self-report* OR emote*)
**R**esearch type	Published studies in English OR Spanish language

- **D**esign: qualitative studies of any design including phenomenology, grounded theory, ethnography, action research and feminist research. Mixed methods design studies where qualitative data can be extracted separately will also be considered for inclusion, as well as survey designs with open-ended questions that provide qualitative data.

- **E**valuation: inductive themes representative of women’s views and experiences of AOL with synthetic oxytocin.

- **R**esearch type: published qualitative studies, in English or Spanish language.

### Search strategy and study selection

An initial scoping search of MEDLINE and CINAHL was undertaken to identify potentially relevant studies and search terms. Search terms were developed using the SPIDER acronym and adapted for each database ( Table 1). The databases to be searched are MEDLINE, CINAHL, EMBASE, PsycINFO, Maternity and Infant Care and Web of Science Core Collection. We will expand our search by additionally searching the grey literature in thesis repositories (EThOS and DART-Europe) and the WHO International Clinical Trials Registry Platform. The reference lists of the studies identified for inclusion will also be reviewed. 

No time restrictions will be applied, and studies published in English or Spanish will be included. Following a search of each database, all citations retrieved will be uploaded into EndNote (version EN20) and duplicates removed. The remaining records will be uploaded to Covidence software for eligibility screening. Titles and abstracts will be screened by two independent reviewers (SAP and DOM) against the inclusion criteria. Full texts of potentially relevant studies will be reviewed. Reasons for exclusion of full text of papers that do not meet the inclusion criteria will be recorded and reported in the full report. Any disagreements that arise between the reviewers at each stage of the screening process will be resolved through discussion, or with an additional review author (DD) until consensus is achieved. The results of the search and the study inclusion process will be reported in full in the final QES and presented in a Preferred Reporting Items for Systematic Reviews and Meta-analyses (PRISMA) flow diagram ( Page *et al.*, 2021).

### Assessment of methodological quality

Appraising methodological quality of the studies included in a QES can strengthen its comprehension and applicability. There are many appraisal tools available and researchers might decide which to use according to their objectives, expertise, time and resources ( Majid & Vanstone, 2018). In this review, we have chosen the Evidence for Policy and Practice Information (EPPI) and Co-ordinating Centre’s appraisal tool. This tool assesses the quality of the methods and the study reporting across 12 criteria ( Table 2). It was designed specifically for synthesising qualitative data ( Thomas *et al.*, 2003) and has been successfully used in previous QES on maternal health ( Panda *et al.*, 2018; Smith *et al.*, 2021).

**Table 2.** Criteria for methodological quality assessment ( Thomas *et al.*, 2003).

*Quality of study reporting*	A - Aims and objectives clearly reported
	B - Adequately described the context of the research
	C - Adequately described the sample & sampling methods
	D - Adequately described the data collection methods
	E - Adequately described the data analysis methods
*There was good or some* *attempt to establish the:*	F - Reliability of the data collection tools
	G - Validity of the data collection tools
	H - Reliability of the data analysis
	I - Validity of the data analysis
*Quality of the methods*	J - Used the appropriate data collection methods to allow for expression of views
	K - Used the appropriate methods for ensuring the analysis was grounded in the views
	L - Actively involved the participants in the design and conduct of the study

Two reviewers will independently quality assess the primary studies (SAP and DOM). The results of the critical appraisal will be reported in narrative and tabular form. All studies, regardless of the results of their methodological quality, will undergo data extraction and synthesis (where possible), as even poorly conducted qualitative studies may provide important data to our QES.

### Data extraction and synthesis

Data will be extracted using a purposively designed data extraction form ( Alòs-Pereñíguez *et al.*, 2021). The data extracted will include specific details about the setting, study period, aim, design, description of the population, methods of data collection and analysis, and findings regarding women’s views and experiences of AOL with synthetic oxytocin. Regarding the findings, data from both, the results and discussion sections will be included. When synthesising the data from the studies included, we will use the thematic synthesis approach developed by Thomas & Harden (2008). This method enables description of recurring themes in the primary studies, as well as creating new concepts and hypotheses. Following the authors’ guidance, the synthesis will be performed in three sequential stages that may overlap to some degree: 1) line by line coding of data from primary studies, 2) development of descriptive themes and 3) generating analytical themes. Coding will be conducted independently by SAP and one other review author (DOM and DD). Subsequently, similarities and differences will be identified, and codes will be grouped into descriptive categories and, through their further interpretation, reflection and discussion within the review team analytical themes will be generated.

### Assessing confidence in the findings

The level of confidence in the review findings will be established using Grading of Recommendations Assessment, Development and Evaluation-Confidence in the Evidence from Reviews of Qualitative research (GRADE-CERQual) ( Booth *et al.*, 2018; Colvin *et al.*, 2018; Glenton *et al.*, 2018; Lewin *et al.*, 2018a; Lewin *et al.*, 2018b; Munthe-Kaas *et al.*, 2018; Noyes *et al.*, 2018). GRADE-CERQual assesses, individually, every distinct review finding according to its four components: the methodological limitations, coherence, extant or adequacy of contributing data, and relevancy to the review question. Then, an overall assessment of confidence in each finding is categorised as high, moderate, low, or very low confidence ( Table 3). Findings will be deemed to be of ‘high confidence’ at the outset and will be downgraded accordingly if there are concerns regarding any of the GRADE-CERQual components. Following the GRADE-CERQual recommendations, this process will be performed independently by two reviewers (SAP and DOM), facilitating opportunities for reflection and discussion within the review team ( Lewin *et al.*, 2018a).

**Table 3.** GRADE-CERQual assessment ( Lewin *et al.*, 2018b).

Level of confidence	Definition
High	It is highly likely that the review finding is a reasonable representation of the phenomenon of interest
Moderate	It is likely that the review finding is a reasonable representation of the phenomenon of interest
Low	It is possible that the review finding is a reasonable representation of the phenomenon of interest
Very low	It is not clear whether the review finding is a reasonable representation of the phenomenon of interest

### Study status

A total of 9306 articles were retrieved and title and abstract screening is in progress. The review will be finished in February 2022.

## Discussion

This QES will be the first to date to synthesise qualitative research on women’s views and experiences of AOL with synthetic oxytocin after spontaneous onset of labour. Due to the frequency with which AOL is carried out in modern maternity care, it is vital that midwives, obstetricians, and policy makers have a clear understanding of how women experience this intervention. The findings of this review will provide a deeper insight into the current gaps in clinical practice. This evidence will potentially support the development of new strategies to improve women’s care into the future.

We will disseminate the findings of this QES through publication in a peer-reviewed journal that predominantly publishes maternity care research and through academic conference presentations. Social media posts (e.g. Instagram and Twitter) will be also employed as part of the dissemination strategy.

## Reflexivity statement

In accordance with rigour in qualitative research, it is important that the authors discuss how their background or expertise may have influenced the interpretation of the findings ( Barrett *et al.,* 2020). SAP is a PhD student with a clinical background in midwifery. She became interested in AOL while working in acute maternity settings, where she observed and performed this intervention. Augmentation of labour has become her PhD topic. DOM, Lecturer in Midwifery, is a general nurse, midwife and clinical midwife teacher. DOM has experience in both quantitative and qualitative methodologies, including expertise in QES.

DD is an Associate Professor in Midwifery. Her areas of methodological research expertise include cohort studies, mixed methods studies, qualitative research, and randomised trials. DD has conducted previous research on the use of synthetic oxytocin during labour. She led a multinational study that highlighted the variations in the regimens and dosages on synthetic oxytocin use.

Acknowledging that the findings of this QES will be the research teams’ interpretation, several procedures will be put into practice to ensure trustworthiness, i.e two independent coders, keeping a research diary which will document thoughts and direction from the beginning of the process of undertaking the QES, and frequent discussions within the research team to agree evolvement of descriptive themes to analytical themes. To add to the transparency of the analytic process, quotations from the primary studies to support the results will be provided.

## Data availability

### Underlying data

No data are associated with this article.

### Extended data

Open Science Framework: Women’s views and experiences of augmentation of labour with synthetic oxytocin. A protocol for a qualitative evidence synthesis, https://doi.org/10.17605/OSF.IO/2QYJC ( Alòs-Pereñíguez *et al.*, 2021).

This project contains the following extended data:

Appendix 1: Data.Extraction.Form.Excel.

### Reporting guidelines

Open Science Framework: PRISMA-P Checklist for ‘Women’s views and experiences of augmentation of labour with synthetic oxytocin infusion. A protocol for a qualitative evidence synthesis’: https://doi.org/10.17605/OSF.IO/2QYJC.

Data are available under the terms of the Creative Commons Attribution 4.0 International license (CC-BY 4.0).
