# Supplementary material for: Women’s views and experiences of augmentation of labour with synthetic oxytocin infusion. A protocol for a qualitative evidence synthesis
Source: HRB Open Res. 2022 Apr 20;4:127. Originally published 2021 Dec 9. [Version 2] doi: 10.12688/hrbopenres.13467.2 (PMC8822135; doi:10.12688/hrbopenres.13467.2)
Supplement: Peer review history

# Peer review: Reviewer response for version 2

**DOI:** 10.21956/hrbopenres.14777.r31889

**Published:** 2022-04-27

**Authors:** Linda Biesty

**Affiliations:** 1 School of Nursing and Midwifery, National University of Ireland Galway, Galway, Ireland

No further comments to add. Wishing the team the best of luck with their QES. I am looking forward to reading the completed work.

Is the study design appropriate for the research question?

Yes

Is the rationale for, and objectives of, the study clearly described?

Yes

Are sufficient details of the methods provided to allow replication by others?

Yes

Are the datasets clearly presented in a useable and accessible format?

Yes

Reviewer Expertise:

My research areas of interest lie in all aspects of midwifery and maternity care. My methodological areas of expertise are qualitative research methodologies and methods including qualitative evidence synthesis.

I confirm that I have read this submission and believe that I have an appropriate level of expertise to confirm that it is of an acceptable scientific standard.

# Peer review: Reviewer response for version 1

**DOI:** 10.21956/hrbopenres.14678.r31348

**Published:** 2022-02-07

**Authors:** Linda Biesty

**Affiliations:** 1 School of Nursing and Midwifery, National University of Ireland Galway, Galway, Ireland

This is an incredibly important study and I am delighted to read the protocol. Some minor points of thought that the review team might consider:

- In the Introduction interesting information is provided in relation to the AOL ranges, variation across criteria, services etc. have the team considered how these variations may impact on their synthesis? 
- In the assessment of methodological quality have the team considered how they will interpret Criteria R,G,H and I, given that the prompts of the criteria speak to reliability and validity?
- The extraction of the "findings" - the team should indicate if they will include findings from findings / results sections of included studies, from discussion section or both? 
- I suggest that it is important that the team include a reflexive section in their protocol to highlight how their professional and research backgrounds will influence their engagement with all methods of this QES. 

Is the study design appropriate for the research question?

Yes

Is the rationale for, and objectives of, the study clearly described?

Yes

Are sufficient details of the methods provided to allow replication by others?

Yes

Are the datasets clearly presented in a useable and accessible format?

Yes

Reviewer Expertise:

My research areas of interest lie in all aspects of midwifery and maternity care. My methodological areas of expertise are qualitative research methodologies and methods including qualitative evidence synthesis.

I confirm that I have read this submission and believe that I have an appropriate level of expertise to confirm that it is of an acceptable scientific standard.

# Response

**Published:** 2022-03-31

**Authors:** Silvia Alòs Pereñíguez

**Affiliations:** Trinity College Dublin, Ireland

Thank you for considering our protocol and giving us some comments and suggestions that will add to the quality of this QES. We have addressed each comment in the table below and highlighted changes in the text of the manuscript.

- *In the Introduction interesting information is provided in relation to the AOL ranges, variation across criteria, services etc. have the team considered how these variations may impact on their synthesis? *
- **Response:** Our QES have included 25 studies providing qualitative data on women’s views and experiences of AOL with synthetic oxytocin infusion from 14 different countries. Of these, some have high AOL rates, others not. This has emerged in our preliminary findings. For example, some women expected to be augmented during labour, while others did not anticipate an AOL and did not read/seek information about it. We feel that the large and varied dataset will ensure that variation rates of AOL will not negatively impact on our QES. Furthermore, assessing the quality of our review findings using CERQual and, in particular, the ‘adequacy and relevance’ components will further address any concern about variations in prevalence of AOL in different jurisdictions.
- *In the assessment of methodological quality have the team considered how they will interpret Criteria R,G,H and I, given that the prompts of the criteria speak to reliability and validity?*
- **Response: **Regarding the methodological quality appraisal, we agreed to use the different criteria as explained in the original article (Thomas *et al.* 2003). For example, regarding the “Reliability of the data collection tools” (F), we considered they met the criteria if the authors used a topic guide or they provided examples of the topics addressed during the interviews. For “Validity of the data collection tools” (G), any type of recording was considered sufficient.  Regarding “Reliability of the data analysis” (H), we considered the criteria met if the more than one author participated in the data analysis and/or if the authors contacted an additional person with qualitative expertise to review the data analysis. We also considered the amount of data provided to support their findings. Regarding “Validity of the data analysis” (I), the criteria was met if the authors conducted negative cases analysis and/or member checking of the preliminary findings.
- *The extraction of the "findings" - the team should indicate if they will include findings from findings / results sections of included studies, from discussion section or both? *
- **Response: **We have included data from both results and discussion sections. Added: 'Regarding the findings, data from both, the results and discussion sections will be included'.
- *I suggest that it is important that the team include a reflexive section in their protocol to highlight how their professional and research backgrounds will influence their engagement with all methods of this QES.*
- **Response: **Added. Please, refer to section “Reflexivity statement”.

# Peer review: Reviewer response for version 1

**DOI:** 10.21956/hrbopenres.14678.r31296

**Published:** 2022-01-31

**Authors:** Claire Feeley

**Affiliations:** 1 King's College London, London, UK

Thank you for giving me the opportunity to review this timely and important qualitative systematic review regarding women's experiences of AOL. It is a very exciting piece of work and I look forward to reading the findings.

 Some minor but important comments:

- **Introduction: **
  - Wouldn't agree 'traditionally' is the right term, maybe typically? Although in the US they are known for AOL with membranes intact...!
  - Need some further acknowledgement/discussion around the huge variation of rates of AOL, why this might be, what does the literature say? That you are focusing on spontaneous labor, this is very important to consider. Birth setting/planned place of birth and where labor is mostly experienced for example will influence the rates of synthesis. Perhaps a minor mention of non-pharma methods e.g. water immersion reduces transfer rates from homebirth/FMU, which haven't had much look in via research? A great opportunity to critique these statistics. 
  - Negative experiences may also mean traumatic experiences/PTSD a point that asserts the importance of getting labor care right so would add this in. (It also influences women to opt out of maternity care altogether, a key point for maternity services to consider).
- **Search strategy:**  What sort of grey literature, be specific.
- **Quality appraisal: **Not convinced this tool is appropriate for qualitative papers, much debate out there appraising quality in qualitative reviews but I personally agree it should be done. However, this tool reads as a quant tool- reliability/validity are positivist terms, you want to look for trustworthiness. Strongly suggest changing this.

Is the study design appropriate for the research question?

Yes

Is the rationale for, and objectives of, the study clearly described?

Yes

Are sufficient details of the methods provided to allow replication by others?

Yes

Are the datasets clearly presented in a useable and accessible format?

Not applicable

Reviewer Expertise:

Midwifery practice, women's experiences of care, midwives supporting women outside of the guidelines, normal birth across the risk spectrum

I confirm that I have read this submission and believe that I have an appropriate level of expertise to confirm that it is of an acceptable scientific standard.

# Response

**Published:** 2022-03-31

**Authors:** Silvia Alòs Pereñíguez

**Affiliations:** Trinity College Dublin, Ireland

**Reviewer comment**

**Reply**

- *Introduction: Wouldn't agree 'traditionally' is the right term, maybe typically? Although in the US they are known for AOL with membranes intact...!*
- **Response: **Yes, we agree. Typically might be more appropriate.
- *Need some further acknowledgement/discussion around the huge variation of rates of AOL, why this might be, what does the literature say? That you are focusing on spontaneous labor, this is very important to consider. Birth setting/planned place of birth and where labor is mostly experienced for example will influence the rates of synthesis. Perhaps a minor mention of non-pharma methods e.g. water immersion reduces transfer rates from homebirth/FMU, which haven't had much look in via research? A great opportunity to critique these statistics. *
- **Response: **Although we acknowledge the importance and the effectiveness of non-pharmacological methods for AOL, this QES will focus on AOL with synthetic oxytocin infusion. Our intention for the full report is to include a paragraph about other non-pharmacological methods that have demonstrated to be effective in managing prolonged labour (i.e, water immersion).Previous research suggested a potential overuse in clinical practice. Further discussion will be included in the QES. Added:
  - […] suggesting a potential misuse in clinical practice.
  - As briefly discussed in the protocol, probably one of the main contributory causes to this disparity is that there is no clear diagnosis criteria for labour dystocia. 
  - […] the lack of consensus on the diagnostic criteria for labour dystocia has brought into question the robustness of its diagnosis.
  - Our preliminary findings also suggest the need to improve the indications for AOL. This will be also discussed in our QES.
- *Negative experiences may also mean traumatic experiences/PTSD a point that asserts the importance of getting labor care right so would add this in. (It also influences women to opt out of maternity care altogether, a key point for maternity services to consider).*
- **Response: **Yes, birth trauma is an important health problem that needs to be considered when talking about childbirth experiences. Added: 'Moreover, women with negative childbirth experiences are more vulnerable to developing serious mental health issues, including posttraumatic stress disorder (Ertan et al. 2021)'
- *Search strategy:  What sort of grey literature, be specific.*
- **Response: **Added: 'We will expand our search by additionally searching the grey literature in thesis repositories (EThOS and DART-Europe) and the WHO International Clinical Trials Registry Platform. The reference lists of studies identified for inclusion will also be reviewed'.
- *Quality appraisal: Not convinced this tool is appropriate for qualitative papers, much debate out there appraising quality in qualitative reviews but I personally agree it should be done. However, this tool reads as a quant tool- reliability/validity are positivist terms, you want to look for trustworthiness. Strongly suggest changing this.*
- **Response: **Although the terms reliability and validity may be considered as quantitative terms, Evidence for Policy and Practice Information (EPPI) and Co-ordinating Centre’s appraisal tool was designed specifically for synthesising qualitative studies (Thomas et al. 2003). It assesses the studies through 12 criteria and has been successfully used in previous QES on maternal health (Panda *et al.* 2018, Smith *et al.* 2021a, Smith *et al.* 2021b)
